# Fiber-Optic-Based System for High-Resolution Monitoring of Stretch in Excised Tissues

**DOI:** 10.3390/bios13100900

**Published:** 2023-09-22

**Authors:** Antonio Velarte, Aranzazu Otin, Pablo Giménez-Gómez, Xavier Muñoz-Berbel, Esther Pueyo

**Affiliations:** 1 Biomedical Signal Interpretation and Computational Simulation (BSICoS) Group, I3A Institute, IIS Aragón, University of Zaragoza, 50018 Zaragoza, Spain; epueyo@unizar.es; 2 Grupo de Electrónica de Potencia y Microelectrónica (GEPM) Group, I3A Institute, University of Zaragoza, 50018 Zaragoza, Spain; aranotin@unizar.es; 3 Department of Materials and Environmental Chemistry, Stockholm University, 106 91 Stockholm, Sweden; pablo.gimenez-gomez@mmk.su.se; 4 Instituto de Microelectrónica de Barcelona (IMB-CNM), Consejo Superior de Investigaciones Científicas (CSIC), Campus UAB, 08193 Cerdanyola del Vallès, Spain; xavier.munoz@imb-cnm.csic.es; 5 Centro de Investigación Biomédica en Red (CIBER) de Bioingeniería, Biomateriales y Nanomedicina (CIBER-BBN), 28029 Madrid, Spain

**Keywords:** biosensors, stretch sensor, excised tissues, POF, cardiac

## Abstract

Cardiovascular diseases cause a high number of deaths nowadays. To improve these statistics, new strategies to better understand the electrical and mechanical abnormalities underlying them are urgently required. This study focuses on the development of a sensor to measure tissue stretch in excised tissues, enabling improved knowledge of biomechanical properties and allowing greater control in real time. A system made of biocompatible materials is described, which is based on two cantilevered platforms that integrate an optical fiber inside them to quantify the amount of stretch the tissues are exposed to with a precision of μm. The operating principle of the sensor is based on the variation of the optical path with the movement of the platforms onto which the samples are fixed. The conducted tests highlight that this system, based on a simple topology and technology, is capable of achieving the desired purpose (a resolution of ∼1 μm), enabling the tissue to be bathed in any medium within the system.

## 1. Introduction

Cardiovascular diseases (CVDs) are the primary cause of death globally [1]. Understanding the electrical and mechanical abnormalities that occur in diseased hearts may lead to improved CVD treatment strategies [2,3,4,5]. In particular, the evaluation of the mechanical stretch of cardiac tissue can be highly relevant in a wide variety of applications [6]. One possibility for measuring cardiac tissue stretch was proposed in [7], where the myocardial slice length was digitally monitored using a camera and 1 mm graph paper attached to the back of the culture chamber throughout stretching. This option lacks the essential resolution, requires camera-attached microscopes, and implies advanced analysis and calibration to provide quantitative stretch measurements. Bioelectronics can play an important role in developing systems and devices that allow for a high level of control by integrating sensing, detection, and real-time responsiveness, particularly in relation to stretch, both in large- and small-scale systems [8,9,10].

One of the most common ways to measure deformation is by using a strain gauge. This type of sensor is based on the variation of the electrical resistance as a function of the stretch or compression applied, which is a very simple and effective technique [11]. Despite its widespread use and simplicity, this type of sensor shows high sensitivity to changes in temperature and interference, which has led to the emergence of other methods [12]. In particular, methods have been developed based on the use of optical fibers, which, in addition to being a transmission medium, can be used as a sensor element. This approach takes advantage of optical fiber features like immunity to electromagnetic interference, compactness, and reliability in hostile environments [13].

Optical-fiber-based sensors have commonly used a technology known as Fiber Bragg Grating (FBG), with a photo-inscribed grating in the core of a single-mode fiber that allows the reflection of some particular wavelengths and the transmission of the others [14,15]. This technique has become increasingly used in recent years in various scientific fields, although new alternatives are still being investigated due to the high sensitivity to temperature, which produces changes in the refractive index and, as a consequence, errors in the results [16].

This work presents an alternative to the use of FBG to perform the accurate real-time evaluation of tissue stretch consisting of an optical-fiber-based sensor manufactured using biocompatible materials. The proposed microsystem is fabricated in polymethylmethacrylate (PMMA) and includes optical elements for ultrasensitive stretch analysis. The system is designed to couple the light and guide it through the optical fiber to the sensor, which is aligned with the collecting optical fiber. The displacement of this fiber in response to mechanical loads or forces applied to the tissues, which are in close contact with the fiber, increases the optical losses of the optical microsystem. These losses are used to monitor mechanical forces in real time.

## 2. Stretch Measurement

### 2.1. Operating Principle

**Figure 1:**
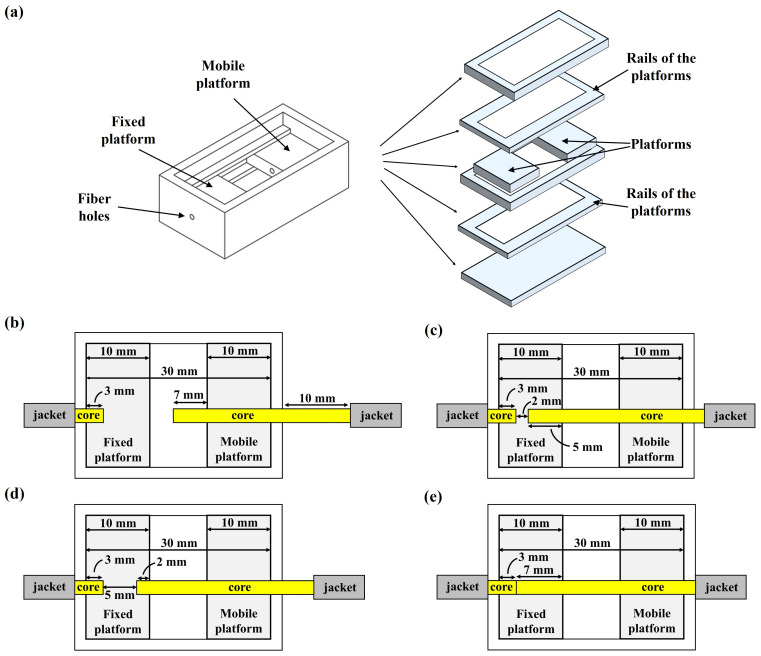
(**a**) Three-dimensional model of the designed PMMA-based structure, including all the two-dimensional layers manufactured with the laser writer; (**b**) Schematic view of the central section from the top of the structure, showing the configuration of the platforms and the arrangement of the embedded fibers; (**c**) Scheme of the initial position of the platforms for the experiments with the tissue; (**d**) Scheme of the platforms positions in the case of the tissue maximum stretch situation; and (**e**) Scheme of the platforms’ positions in the case of maximum tissue shrinking.

A structure of PMMA was designed with Corel Draw v.20 software and manufactured using a CO${}_{2}$-laser printer (Epilog Mini 24, Epilog Laser, Golden, CO, USA). PMMA was selected because of its easy manufacturing, robustness, low cost, and biocompatible nature. The structure was formed using five two-dimensional layers of PMMA bonded with double-layered pressure sensitive adhesive (PSA) to obtain the three-dimensional structure presented in Figure 1a. The assembled PMMA-based structure (34 mm length, 18 mm width, 11 mm height) was hollow, with a wall thickness of 2 mm. A 5 mm PMMA layer on the bottom (34 mm length, 18 mm width, 4 mm height) served as support for the rest of the structure. Two PMMA-based platforms (10 mm length, 10 mm width, 2 mm height) with a 1 mm diameter cylindrical hold in the middle were integrated at half the height of the structure, held by PMMA rails (10 mm length, 18 mm width, and 1 mm height, with an inner hollow of 10 mm × 14 mm), bonded to the bottom and top layers of the structure. The platform was completed by a 2 mm PMMA layer on the top bonded to the top rail using PSA. One of the platforms was fixed to the rails, while the other platform was able to move freely through it in response to mechanical loads. Two optical fibers (MH4001 EskaTM, 1.0 mm Core Simplex High-Performance Plastic Optical Fiber, 2.2 mm OD Polyethylene Jacketed, Industrial Fiber Optics, Tempe, AZ, USA) were embedded into both 1 mm diameter holes, located in the middle of each platform (Figure 1b). For the fixed platform, the optical fiber was embedded 3 mm, leaving the remaining 7 mm of the hole unoccupied. For the mobile platform, the optical fiber crossed the entire length of the platform (10 mm), protruding 7 mm on the other side of the platform. The embedded optical fiber in the fixed platform and the one embedded in the mobile platform were connected to an optical transceiver (Analog OptoLock^®^ FC300T, Laser Components^®^, Olching, Germany) that included a light source and a photodiode to collect the light that reached the other side of the fiber.

The aim of the structure was to measure small deformations in cardiac tissues as a function of the light fraction reaching the receptor, which depended on the distance between both fibers [9] (i.e., the intensity that reached the receiving fiber changed according to the separation between both platforms). In the initial position (i.e., the tissue placed on the top of both platforms), the mobile platform was displaced 8 mm from the right of the structure, and the emitting optical fiber entered 5 mm in the fixed platform. This resulted in a distance between both fibers of 2 mm (Figure 1c). If the tissue stretched, the mobile platform was displaced to the right, increasing the distance between the fibers to 5 mm in the case of the tissue’s maximum stretch situation (Figure 1d), causing a decrease in the light fraction reaching the receptor in comparison to the initial position. On the other hand, if the tissue shrank, the mobile platform displaced to the left, and the distance between the fibers decreased, causing the fibers to touch each other in the case of maximum shrinking (Figure 1e), and increasing the light fraction reaching the receptor.

### 2.2. Validation through Simulation

The effect of the change in the distance between the optical fibers integrated into the designed PMMA device was evaluated by using the computational COMSOL Multiphysics^®^ 6.0 software (COMSOL). The Electromagnetic Waves Beam Envelopes physics Interface was used for the simulation. The selected interface enabled the computation of the electric and magnetic field distributions of the device because the amplitude varied slowly on the wavelength scale. The electric field (*E*) was factored into a slowly varying electric field envelope function ($E_{1}$) and a rapidly varying phase function ($\varphi_{1}$(*r*)) according to the following equation:

(1) $$E\left(r\right)=E_{1}\left(r\right)e^{-j\varphi_{1}\left(r\right)}$$

Equation (1) combined with Maxwell’s equation results in the wave equation for the envelope function:

(2) $$\left(▽-jk_{1}\left(r\right)\right)\times \left(\left(▽-jk_{1}\left(r\right)\right)\times E_{1}\left(r\right)\right)-k^{2}E_{1}\left(r\right)=0$$

where $k_{1}\left(r\right)=▽\varphi_{1}$ and *k* is the wave number, expressed as *k* = $k_{0}$n, which depends on the refractive index (n) and the number of free space $k_{0}=\omega /c_{0}$, where $c_{0}$ is the speed of light in a vacuum.

The electric displacement field, $\varepsilon_{r}$, depended on the refractive index of each material: $\varepsilon_{r}=(n\;$–${\;ik)}^{2}$.

**Figure 2:**
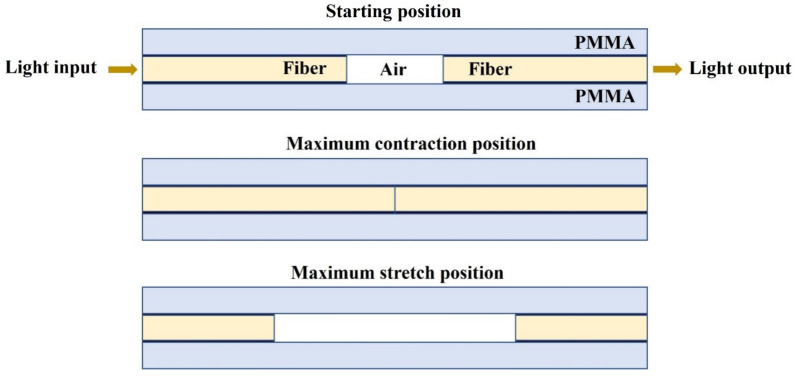
Two-dimensional scheme of device cross-section used for the simulations for the initial, maximum contraction, and maximum stretch positions.

The platform integrating the fibers was simulated in a 2D design to predict the transmittance at the output of the receiving filter as a function of the distance between the PMMA platforms in which the fibers were placed (Figure 2). The fibers were placed into two symmetric PMMA platforms and initially separated by a distance of 2 mm, which decreased or increased when the tissue contracted or stretched, respectively. The following parameters were defined for all the simulations: cladding width of 3 mm, core width of 2 mm, fiber length of 10 mm, core refractive index of 1.51, cladding refractive index of 1.48 (PMMA), air refractive index of 1, and port input power of 0.021 W/mm. The best results were obtained with the electric field components solved using the out-of-plane vector and the unidirectional wave vector. Moreover, a physically controlled mesh was applied for the simulations. The influence of the recorded transmittance in the output of the receiving fiber was assessed by using distances between 0 mm and 5 mm (0.0 mm, 0.5 mm. 1.0 mm, 2.0 mm, 2.5 mm, 3.0 mm, 4.0 mm, and 5.0 mm).

**Figure 3:**
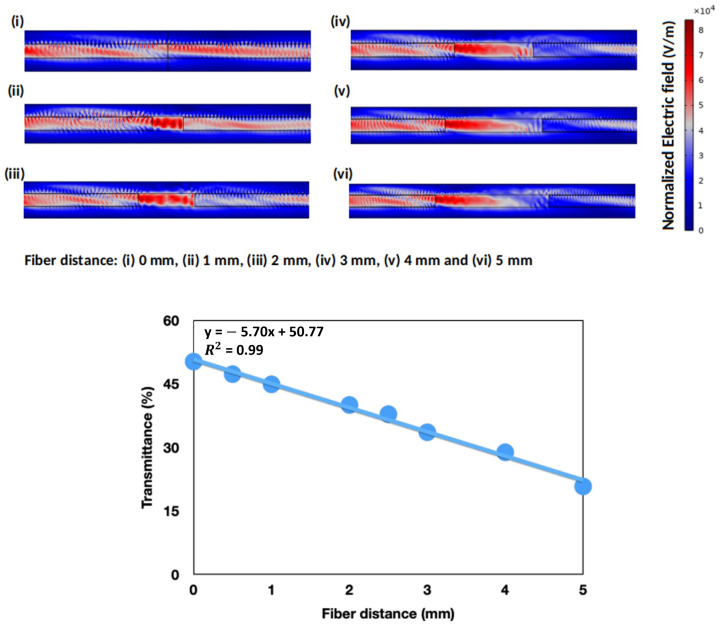
Color scheme of the normalized electric field obtained for a distance of 0 mm (i), 1 mm (ii), 2 mm (iii), 3 mm (iv), 4 mm (v) and 5 mm (vi) between the emitting and the receiving optical fibers. Plotted variation of the calculated transmittance as a function of the fiber distance.

The results obtained from the simulations are summarized in Figure 3. Images from (i) to (vi) represent some of the normalized electric fields obtained using COMSOL, depending on the distance between the optical fibers. The surface color system represents the normalized electric field throughout the device, with dark red, dark blue, and bluish-red for high, low, and intermediate values, respectively. As expected, the electric field reaching the output of the receiving fiber on the right decreased when the distance between fibers increased. These losses were associated with the scattering and dispersion of light when traveling from the light-input optical fiber to the detector. The transmittance for each distance was calculated and plotted in Figure 3, showing a linear relationship between these two variables in the range under study.

## 3. Sensor Prototype

Several prototypes were developed before the final version. Initially, flat platforms that moved along the guides located on the sides of the sensor were used, but there were some issues correctly fixing the fiber position in order to achieve a good optical path alignment. In the next iteration, SMA connectors were included, which allowed us to set the position correctly, but their weight was too high to allow the displacement of the platforms. To solve this issue in the final prototype, a part of the optical fiber was stripped to introduce it into the holes, which allowed us to fix its position and have a low weight. Since the moving end of the fiber never leaves the fixed platform (Figure 1d), the integrity of the optical fiber is ensured and therefore no other protection technique is required. Additionally, we decided to fix one of the sensor platforms and keep it in a static position to achieve more accurate measurements by allowing the movement of only one platform.

### 3.1. Stretch Sensor for Excised Cardiac Tissues

The sensor was designed for biomedical use, specifically for measuring stretch in small excised tissues. To preserve the tissue in physiological conditions, it was ensured that this remained in contact with the culture medium that delivered the required nutrients and proper oxygenation.

**Figure 4:**
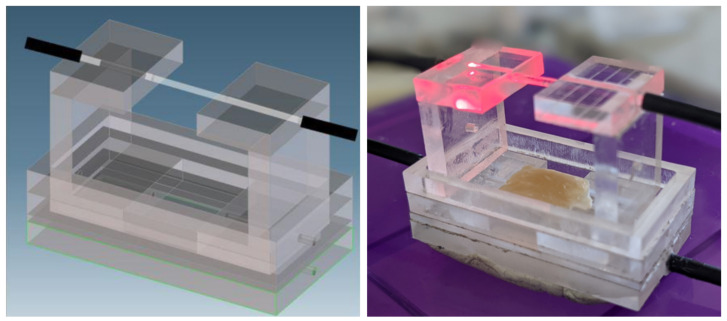
Fiber-optic sensor based on mobile platforms. In the (**left panel**), the 3D model shows the discontinuity between both stripped parts of the optical fiber when they are separated. The fabricated sensor includes the guides along which both PMMA platforms move. In the (**right panel**), the cardiac tissue placed on the sensor to measure the stretch is shown. The tissue sample is located in contact with the medium, which does not influence the stretch measurement since this is based on the upper fibers.

As mentioned above, the first sensor prototype was composed of two flat platforms that incorporated the optical fibers. Consequently, the medium and the fiber were in contact, which rendered results highly dependent on the considered medium. Subsequently, the sensor was modified in such a way that the fibers were located on the top part of the platform (Figure 4). In this case, the behavior of the sensor was independent of the employed medium, and its response only depended on the stretch of the tissue since the fibers moved jointly with the cardiac tissue sample. To ensure this synchrony, the lower part of the sensor had small protrusions designed to position and attach the tissue. Hence, one of the platforms was displaced a distance equal to the stretch experienced by the tissue. Since, with this modification, the transmission medium of the light emitted by the optical fiber was always the same (i.e., the air contained in the cavity), it allowed us to carry out experiments with different culture media without requiring either variations in the electronic system or adjustments of the output signal in the post-processing.

### 3.2. Electronic Acquisition System

**Figure 5:**
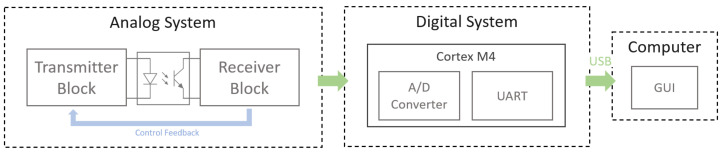
Block diagram of the whole electronic system of the sensor. The electronics are divided into analog and digital zones and have USB communication through which the data are transmitted to the PC, where they are displayed on the graphical user interface (GUI). The blue feedback signal refers to the output signal of the system before it is digitized.

The portable design of the sensor’s electronic system leads to its versatile use in different places such as hospital laboratories or cell/tissue culture rooms. For this purpose, a 5 V power supply was selected for the whole system like a USB port of a laptop. The block diagram of the system is shown in Figure 5, which contains an analog part dedicated to the emission and reception of guided light using an optocoupler, a digital one based on a microcontroller, and a graphical user interface (GUI) to visualize data in real time. The analog part includes a feedback control that automatically adjusts some details of the sensor, such as temperature compensation over time, for which a second optical fiber (with a cut between the ends to give it the same properties as the one that acts as sensing element) has been included at the bottom of the sensor (Figure 4).

Before the electronics were mounted using discrete components, each piece was individually simulated to verify its correct operation. OrCAD^®^ was the software selected to conduct these simulations.

Analog electronics were divided into two main parts, the transmitter and the receiver block, communicated by an optical fiber, which was cut to act as a sensing element dependent on the distance between both. The transmitter and receiver were on the same end, so this system was classified as a transmission configuration, as in the study by Kuang et al. [17]. A plastic optical fiber (POF) with a core of 1 mm thickness was selected due to its properties, including mechanical resistance, accessibility, and ease of manipulation. Specifically, MH4001 Eska™ Mega was used in the sensor, a fiber from Mitsubishi Chemical Corporation.

For the design of the transmitter, an LED diode was chosen as a light source to emit the light signal towards one end of the optical fiber. The corresponding polarization circuit for the transmitter element and a generator of the electrical excitation signal were also designed. The LED selection was based on the results obtained with the spectrometer during preliminary tests, which showed larger variations for wavelengths in the red light region when the optical path was modified (see Section 4.1).

A photodiode was placed at the opposite end of the optical fiber to collect the light crossing the sensor at the different deformation conditions. In this receiver block, in addition to the polarization circuit of the photodiode, an impedance adapter and a second-order low-pass filter with a Sallen–Key topology were included to mitigate the ambient noise. Additionally, the system gain was adjusted to the desired values to perform signal feedback and digitize it. With the introduction of the output signal feedback, it was possible to control some aspects of the luminous excitation electronics, such as the thermal drift or the offset that appeared between the various developed prototypes.

**Figure 6:**
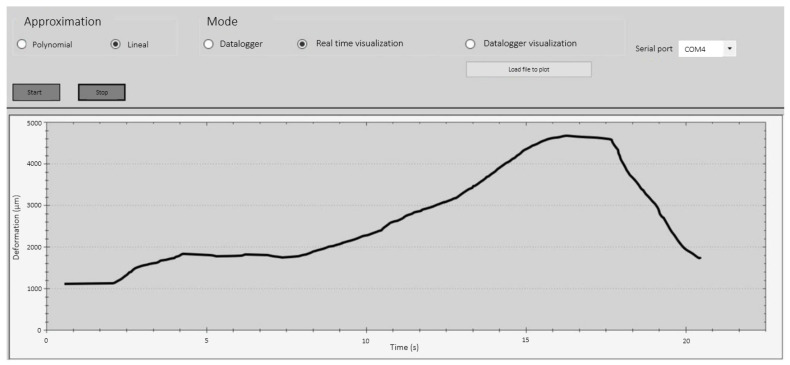
Graphical user interface developed for the sensor system. This example shows a change in the deformation (ordinate) versus time (abscissa) in the data from the COM4 port.

In the first validation tests of the sensor performance, an oscilloscope was used to verify the values obtained at the output, thus allowing us to calculate the corresponding Current Transfer Ratio (CTR) in relation to the known input value. A more portable and compact version was then produced by implementing a CortexM4 microcontroller, which enabled the digitization of the output signal of the system. The on-chip ADC converter, which had 12 bits and an input signal range of 0–3.3 V, was used to make the conversion. The digitized signal was then sent through a USB port via the UART protocol to the PC or the laptop, where the data were displayed on a personalized GUI developed in C#. The GUI had a simple interface and allowed the user to select between real-time signal display, data recording in datalogger mode, and an extra option for graphing saved data. The simplest operation flow consisted of selecting the serial port (COM) associated with the sensor board and pressing the start button. An example of real-time operation is shown in Figure 6.

### 3.3. Experimental Characterization: Procedure and Setup Configuration

**Figure 7:**
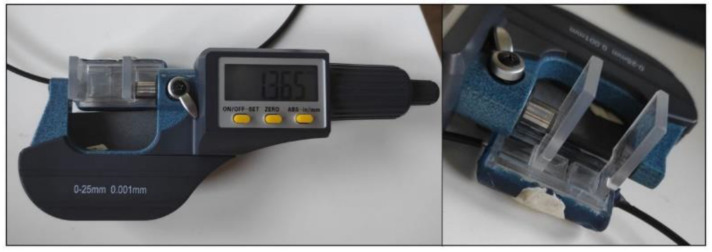
Setup used for sensor characterization. A digital micrometer is used to relate the intensity obtained from the electronic measurement instrumentation with the displacement of the fibers, to establish the system’s behavior.

To characterize the sensor performance, several experiments were carried out with the setup presented in Figure 7. The setup consisted of the sensor, a digital micrometer with a resolution of 1 μm and an accuracy of ±2 μm; the custom-made electronic instrumentation system and an oscilloscope were used to measure the response or output signal of the system, as well as to verify that the complete system worked as expected.

The procedure carried out to characterize the system involved the adjustment of the distance between the two fiber cores and the recording of the optical signal. The initial situation considered the two fibers to be separated 5 mm in an approach test and 0 mm in a separation test. The values were selected based on the characteristics of the excised tissue samples [18]. The sensor was then characterized considering that the samples typically experienced deformations of up to 20%.

The characteristic index CTR, defined as the relation of the intensities flowing through the photodiode and the LED, was obtained for each experiment. Since the static CTR showed a good correlation with the stretch of the tissue, this index was employed and the dynamic CTR was discarded.

After several complete sweeps for the displacement were performed while using the three different prototypes, statistical results were obtained. Some tests were also performed with different culture media to assess its influence. Furthermore, a series of experiments were performed by displacing the fibers at random distances, and the operation of the sensor was verified.

## 4. Results and Discussion

### 4.1. Optocoupler Pair Selection

**Figure 8:**
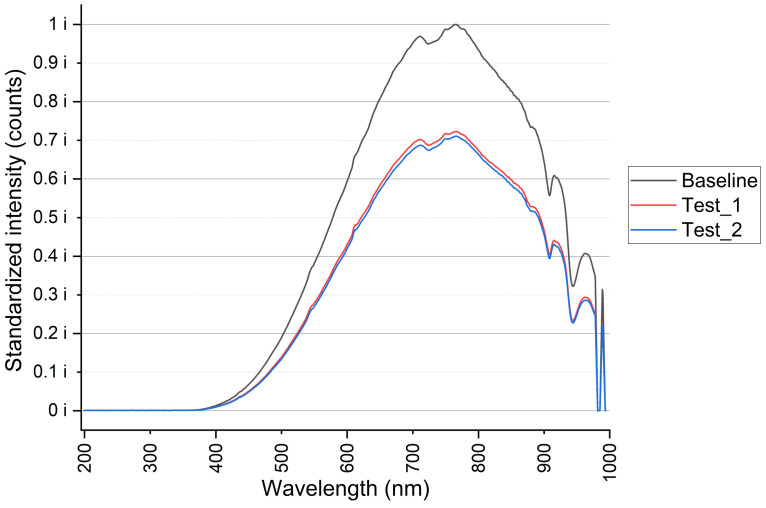
Optical path characterization curve. Gray: initial situation; fibers are close. Red, blue: maximum displacement tests (worst-case situation).

First, optical spectroscopy measurements were performed with a broadband visible light source HL-2000 (Ocean Optics) and the QE6500 spectrometer (Ocean Optics) to determine the optimal wavelength for sensor operation. Figure 8 shows the light intensity (standardized) reaching the sensor detector in the UV-VIS wavelength range (from 195 to 1000 nm). For the input excitation considered, the maximum received intensity (when the fibers were in contact) was 39,221 counts, the value assumed with 1.0 *i*. To simplify the data, the curve was normalized to this intensity value.

Several measurements were carried out at baseline (fibers in contact), obtaining repetitive values (max standard deviation (SD) = 0.01 *i*). The average of these repeats is shown in gray in the figure. After displacement, the magnitude of light intensity decreased, as shown in the other two lines corresponding to displacement tests of 5 mm.

The peaks of maximum transmission were obtained in the wavelength range between 650 nm and 760 nm, which corresponded to the maximum of the light intensity of the source. A DC300T optocoupler pair from laser components was selected, which works with a wavelength around 660 nm (red) and presents an integrated POF fiber fixation system that simplifies the fibers’ alignments for optimal light coupling.

### 4.2. Sensor Characteristic Curve

To verify the sensor behavior seen in the simulation, an experimental characterization was performed considering the whole range of displacement, from 0 to 5 mm, using the setup described in Section 3.3. Photodiode and LED currents were measured simultaneously with the displacement of the free platform over the entire displacement range. The obtained values were used to compute the static CTR and the curve that relates CTR and the separation distance. Several complete sweeps of the displacement range were performed to verify the repeatability of the sensor.

**Figure 9:**
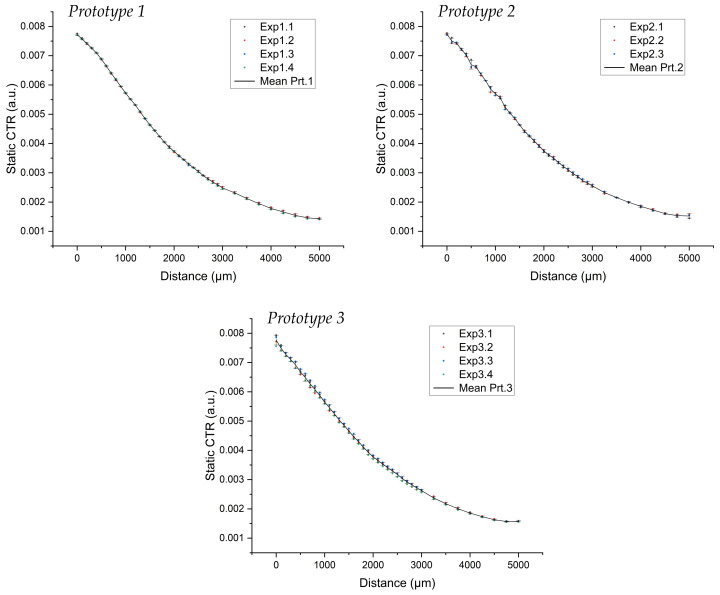
Static CTR as a function of the gap between the two optical fibers. The three subfigures show each prototype, where the measurements were taken for each one, and the calculated mean and SD are shown. The mean curve in black is calculated over all iterations of each prototype.

Figure 9 shows the results obtained when moving the fibers closer from an initial separation of 5 mm. The figure confirms that the response of the sensor was distance-dependent, with lower magnitudes when the two fibers were more separated. The response of the sensor was precise and repetitive, and the curves obtained in the different runs performed with each of the prototypes were all very similar. This enables parallel testing to be conducted under different conditions, which will facilitate the acquisition of reliable data and allow for more detailed studies.

To evaluate the variability of each prototype over iterations, the standard deviation (SD) of the static CTR was calculated for each of them. The results are shown in Figure 9 together with the mean calculated for each prototype. The low SD of all cases indicated that each sensor has low variability over different tests. The average SD values for the different prototypes are $2.41\times 10^{-5}$, $4.32\times 10^{-5}$ and $6.71\times 10^{-5}$, respectively. The maximum value obtained when calculating the SD was in prototype 3, which was $1.69\times 10^{-4}$ for a null gap distance between the fibers. The standard deviation values were slightly worse for shorter distances and presented stable values in the order of $10^{-5}$ once the separation distance reached 1100–1200 mm.

**Figure 10:**
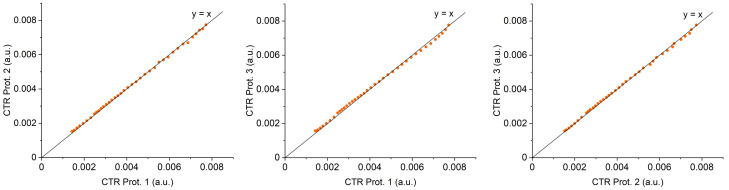
The x-y representation of the data obtained with the different prototypes, with a line of equality. It allows a quick evaluation of the degree of agreement between two prototypes.

To assess the agreement between prototypes, first, an x-y graph including the line of equality (Figure 10) was represented. This graph provided a first view of the relationship between the two variables (the two prototypes compared in our case). The close distance of the points to the diagonal indicates a high degree of agreement.

The Spearman correlation coefficient between the CTR measures for each pair of prototypes was calculated. In all cases, the coefficient values were very close to 1 and with a *p*-value that was always less than 0.05, indicating a high correlation between prototypes.

**Figure 11:**
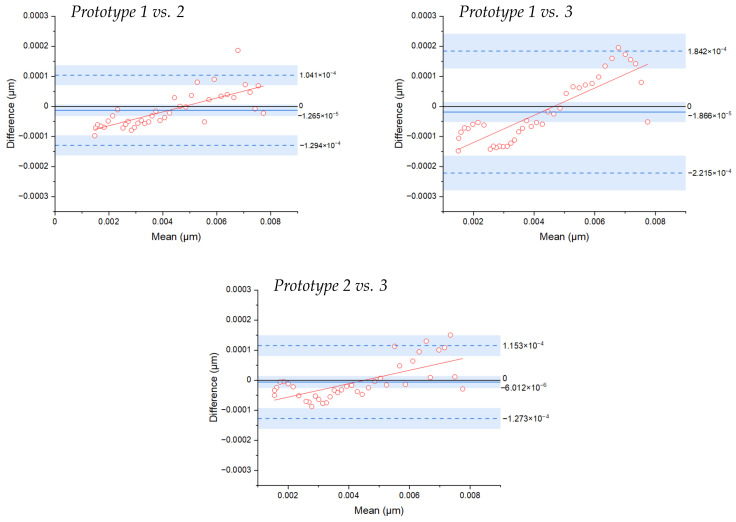
Bland–Altman statistics calculated for each pair of prototypes to assess the agreement between the different analyzed prototypes.

Next, the difference between the methods versus their mean was represented using Bland–Altman (BA) analysis (Figure 11). We observed that the different prototypes had similar behavior, with the mean of the differences at $-1.26\times 10^{-5}$, $-1.86\times 10^{-5}$ and $-6.01\times 10^{-6}$ for each of the three comparisons. The sensor acting as a reference in each case rendered smaller values for smaller measured mean values, which corresponds to the larger separation distances between the fibers that compose the sensor. This analysis demonstrates the high similarity between the different prototypes, thus confirming that the sensor’s behavior was reproducible and rendered highly accurate results.

### 4.3. Random Point Testing

**Figure 12:**
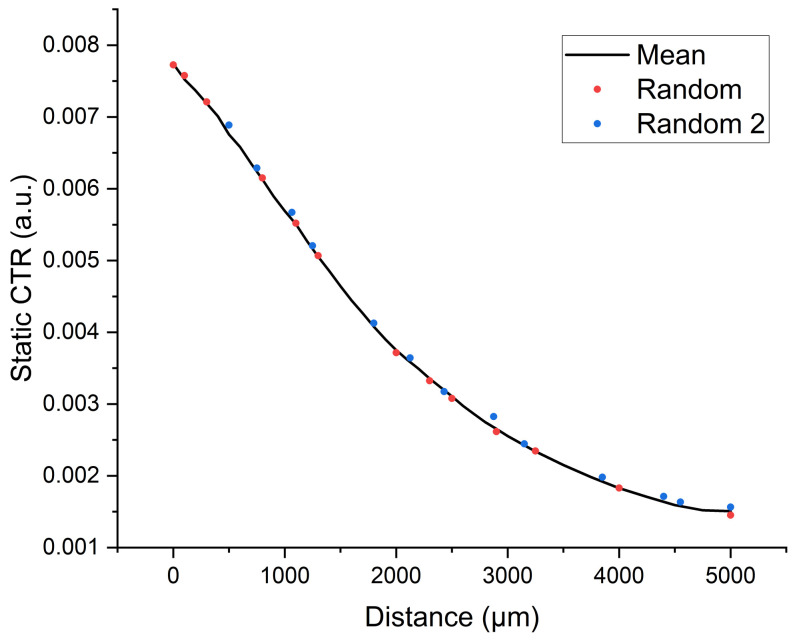
Static CTR for a test performed to verify the behavior of the sensor for random distances. Note that the mean curve represented in this figure is the mean of the three prototype curves.

To verify the behavior of the system in a real situation, a series of tests were performed in which the fibers were displaced following a random separation distance, which were calculated using the Python function random.uniform, which allows one to obtain randomly uniformly distributed numbers between two given values. The response of the system was measured using a digital micrometer, which allowed us to validate that the system behavior was as expected. A sample of these experiments is shown in Figure 12. Red and blue dots represent the values returned by the sensor for the random distances generated by a Python algorithm. The sensor’s behavior was successful, given that the vast majority of values from these random tests were located very close to the sensor’s characteristic curve.

## 5. Conclusions

A fiber-optic-based system made with biocompatible materials has been presented, which enables the evaluation of stretch in excised tissues of small dimensions (max. of 5 mm × 7 mm) with a high resolution (1 μm). The microsystem’s functionality is obtained thanks to a specific design based on a structure composed of two mobile platforms, which allow the sensor to operate independently of the considered electrolyte. This flexibility enables the creation of an experimental bank to correlate the tissue’s stretch degree with several drugs, which has a significant impact on medical and pharmacological research (evaluation of tissue response to different compounds and the optimization of treatments). Compared to existing methods, the proposed sensor system becomes a simpler alternative, in both fabrication and operation, leading to high-resolution measurements, a high level of miniaturization, and compatibility with different culture media, becoming a versatile and powerful tool for biomedical research.

## Data Availability

All the data generated/analyzed in this study are included in this article itself.
